# Acute mental health concerns in emergency settings: development and validation of an Ovid MEDLINE search filter

**DOI:** 10.5195/jmla.2025.2081

**Published:** 2025-07-01

**Authors:** Nicole Askin, Mark Heinrich Mueller

**Affiliations:** 1 Nicole.Askin@umanitoba.ca, Winnipeg Regional Health Authority Virtual Library, University of Manitoba, Winnipeg, MB, Canada; 2 mark.mueller@saskhealthauthority.ca, Saskatchewan Health Authority Clinical Sciences Library, Saskatchewan Health Authority, Regina, SK, Canada

**Keywords:** Search filter validation, search hedge validation, emergencies

## Abstract

**Background:**

The authors sought to develop and validate a search filter to retrieve research about acute mental health concerns during public health emergencies. They did so as a response to a recommendation from a previously published paper on searching for evidence in emergency contexts.

**Methods:**

The definition of acute mental health was adapted from the DSM-5 and the DynaMed entries on acute stress and posttraumatic stress disorder. The definition of public health emergencies was adapted from the Canadian Medical Protective Association. The authors retrieved systematic reviews on mental health concerns pertaining to people in the community and healthcare workers during public health emergencies from MEDLINE. The authors formulated gold standard sets for each population group using articles included in these reviews. The authors then separated the articles into development and validation sets. Keywords and Medical Subject Heading (MeSH) terms from the title and abstracts in the Ovid records in the development sets were used to formulate the filter. The filter was tested via the relative recall method using the validation sets. The authors then tested the filter for precision by conducting MEDLINE (Ovid) searches for the following topics for acute mental health: (i) children/adolescents and earthquakes; (ii) children/adolescents and Ebola outbreaks; (iii) healthcare workers and earthquakes; and (iv) healthcare workers and Ebola outbreaks.

**Results:**

The MEDLINE filter demonstrated 100% recall against the people in the community validation set and 98% recall against the healthcare worker validation set. The filter demonstrated the following percentages for the precision tests: (i) 94% for children/adolescents and earthquakes; (ii) 81% for children/adolescents and Ebola outbreaks; (iii) 81% for healthcare workers and earthquakes; and (iv) 79% for healthcare workers and Ebola outbreaks.

**Conclusion:**

The authors developed a validated search filter that could be used to find evidence related to acute mental health concerns in public health emergencies. The authors recommend that researchers adapt and modify the search filter to reflect the unique mental health issues of their population groups.

## INTRODUCTION

Public health emergencies and catastrophes pose significant psychological threats for many population groups and have the potential to do so in the future (1, 2). Exposure to a public health emergency or a catastrophe is common and one-third or more individuals may develop posttraumatic stress disorder and/or other psychological disorders after severe exposure to the event (3). The COVID-19 pandemic was no exception: several systematic reviews detailed a high prevalence of anxiety, depression, and trauma across affected countries and populations (3–5). One study conducted by Zhou et al suggested that posttraumatic stress disorder was a concerning mental health problem in light of several major emergencies over the past two decades, and that it is crucial to further explore the psychological mechanisms and effective strategies for prevention and intervention (2).

To achieve this goal, researchers need to find ways to optimize their retrieval of evidence, which can be hampered by poor search strategies (6). Finding evidence during a public health emergency, especially in the case of a novel pandemic, poses many challenges. During the COVID-19 pandemic, for instance, many librarians, particularly clinical librarians working out of the hospitals, were tasked with finding answers to questions that seemed to outpace the evidence in a rapidly evolving information environment (7). These challenges included the volume and heterogeneity of the literature being produced, the rapid production of data, and the proliferation of new resources, all of which prompted the Librarian Reserve Corps to draft a white paper on searching for evidence during public health emergencies (7).

Before constructing the filter presented here, we consulted several resources for validated filters for acute mental health that could be applied to public health emergencies. We were unable to find any existing validated filters for this topic on the ISSG Search Filter Resource (20), the Scottish Intercollegiate Guideline Network (21), the Penn Center for Evidence-Based Practice (22), or the University of Alberta Health Sciences Search Filters website. (26) The Ovid Expert Searches website published by Wolters Kluwer contains specific searches for COVID-19 and mental health, coping with fires and wildfires, earthquakes and PTSD (10), but the reference lists and validation data are lacking for many of these filters.

In terms of non-validated searches, we found through an Internet search three search strategies that were limited to mental health in the COVID-19 pandemic and used terms that may not be generalizable to other types of disasters (i.e. quarantine, physical distancing, etc.) (9, 23, 24, 25). Finally, we searched CABI's searchRxiv (29) using the terms “acute mental health,” “disaster mental health,” and “emergency mental health,” and we were unable to find any search strategies related to acute mental health in the public health emergencies context.

The one filter we did find was that of Wilcynski et al (31); however, this study did not use the relative recall method, the gold standard for the validation of a search filter, which we used in our study.

We recognized the value of developing validated filters to assist with responses to the evolving information needs of stakeholders during emergencies (7). This manuscript describes the development and validation of a search filter that can be used to retrieve evidence pertaining to acute mental health conditions during public health emergencies. This validated search filter is designed for use in MEDLINE (Ovid) and does not have a specific methodological focus.

This filter is designed to be paired with emergency-specific filters such as those provided by the CADTH (now Canada's Drug Agency) Covid-19 Search Strings website (8), the COVID-19 Repository (9), the filters for emergencies and disasters on the Ovid Expert Searches website (10), and other filters for emergencies and disasters. It is designed to achieve a balance between recall and specificity, and can be used by clinicians, librarians, policymakers, or public health professionals who are finding evidence during public health emergencies.

## METHODS

### Scope and Definitions

In this project, we designed and validated a search filter to retrieve evidence for acute mental health concerns during public health emergencies. We did not design the filter with the aim of retrieving literature on populations groups with pre-existing mental health conditions. We also did not aim to include studies that focused on the long-term sequelae, prognosis, and treatments for mental health. Our aim was to capture literature that reflected the scope of the United Nations Office for Disaster Risk Reduction (UNDRR) response: actions taken directly before, during or immediately after a disaster to reduce health impacts and “predominantly focused on immediate and short-term needs” (32).

We consulted the following entry for acute stress disorder in the Diagnostic and Statistical Manual of Mental Disorders (DSM-5) to inform our definition of acute mental disorders: “The essential feature of acute stress disorder is the development of characteristic symptoms lasting from 3 days to 1 month following exposure to one or more traumatic events” (33). We are cognizant that the timeframe for when acute stress disorder becomes a chronic condition is a matter of debate among professionals in the mental health community. We also consulted the Acute Stress Disorder entry in DynaMed and searched APA PsycINFO, MEDLINE and the grey literature using the terms “acute stress,” “acute trauma,” “acute mental disorders” and “acute mental health conditions.” We were unable to find consistent alternative definitions for acute stress. We noted that the evaluation criteria for posttraumatic stress disorder in DynaMed included the following: “Symptoms must persist for more than 1 month. Some patients may not meet the full diagnostic criteria until at least 6 months after the event (delayed expression)” (34). We used both definitions in the DSM-5 and DynaMed to capture populations with both immediate and delayed expressions of trauma – that is, mental health symptoms lasting from 3 days to 6 months after the event. The mental health concerns included in the filter were the psychological and emotional symptoms related to traumatic events including posttraumatic stress, anxiety, depression, behavioural and emotional problems, burnout, and compassion fatigue.

We consulted the Canadian Medical Protection Association's (CMPA) Public Health Emergencies and Catastrophic Events page to inform our definition of public health emergencies. The CMPA site defines a public health emergency as: “an urgent and critical situation of temporary nature that seriously endangers the lives, health and/or safety of the population. It would require prompt action beyond normal procedures to prevent or limit health consequences to the affected population” (35). These public health emergencies could include pandemics and disease outbreaks, natural disasters, mass casualty incidents, wars, and political violence.

We also wanted to include the following two broad population groups when curating the development and validation sets: (i) people in the community and (ii) healthcare workers. By “people in the community” we mean any person in the community living and working in non-healthcare settings. By “healthcare workers” we mean any person working in any healthcare context (i.e. physicians, nurses, therapists, etc.). When developing the filter, we hypothesized that these two population groups would require a different search approach due to different perspectives and the degrees to which each would be mentally impacted by the public health emergency (i.e. burnout, compassion fatigue, etc.).

### Creating the Gold Standard Sets

We used the “relative recall” method to validate the search filter. Sampson et al demonstrated the use of the relative recall method in search validation (11). They proposed a collection of 100 relevant studies derived from existing systematic reviews as being an appropriate gold standard for the validation of a search filter. This relative recall method was subsequently adopted by multiple researchers working on filter validation projects (12-14).

For this project, we adapted a published step-by-step guide to creating a validated geographic filter (14) using the relative recall method.

To build our gold standard sets, we began by identifying systematic reviews in MEDLINE on mental health concerns during public health emergencies using a combination of keywords and MeSH to capture the following concepts: (i) mental, psychological, emotional symptoms; (ii) acute symptoms; (iii) pandemics, epidemics, natural disasters, human-made disasters, public health emergencies; (iv) healthcare workers; (v) seniors, adults, adolescents, or children in the community. We aimed to locate a similar number of articles for both healthcare professionals and people in the community. We also aimed to identify reviews addressing a variety of contexts to improve the transferability of the findings. We found that the recent literature focused on the COVID-19 pandemic and infectious diseases in general. Thus, we made a concerted effort to find reviews on natural and human-made disasters ranging from chemical and biological warfare to wildfires. For reviews to be included in the gold standard sets they needed to focus on mental health symptoms lasting from 3 days to 6 months after the event. The reviews also needed to provide a description of a search strategy that at least utilized PubMed or Medline to be considered for inclusion.

We excluded reviews on mental health concerns related to climate change. While climate change is a serious issue and an ongoing emergency it does not present the same acute trauma as other emergencies. Reviews on wildfires, hurricanes, tornadoes, and other weather-related disasters that are connected to climate change were included because they considered the acute mental health concerns in these contexts. We also excluded reviews that focused on individuals with pre-existing mental health conditions, that did not provide details of the search strategy, and that were not indexed in MEDLINE or PubMed.

We gathered 54 reviews on acute mental health concerns during public health emergencies for people in the community and 39 reviews for healthcare workers. The two authors screened these reviews and excluded 32 reviews for people in the community and 17 reviews for healthcare workers because they did not meet the inclusion criteria. The two authors resolved any disputes that arose by consensus. We were left with 22 reviews for people in the community and 22 reviews for healthcare workers. We extracted the included studies from these reviews. This resulted in a total of 525 citations with publication years ranging from 1987 to 2023: 206 articles for people in the community and 319 articles for healthcare professionals. We then divided each collection into two sets: a development set for creation of the filter and assessment of internal validity, and a validation set for assessment of external validity. After randomly ordering the articles, we divided them into the following sets comprising our gold standard: (i) 103 articles for a development set for people in the community; (ii) 103 articles for a validation set for people in the community; (iii) 159 articles for a development set for healthcare workers; and (iv) 160 articles for a validation set for healthcare workers. This process provided us with a sufficient size to allow for a reasonable confidence interval in validation as described by Sampson et al (11). Appendices B and C list the included reviews.

### Development of the Filter

We created the initial filter for Ovid MEDLINE based on the results in the community and healthcare worker development sets. The records were reviewed for search terms reflecting the concept of acute mental health issues. The first author reviewed the articles for people in the community and the second author reviewed the articles for healthcare workers. Both authors extracted broad concepts related to mental health problems (affective symptoms, behavioral and emotional symptoms, mental disorders) and acute symptoms (acute disease, early diagnosis, incidence, prevalence, cohort studies and cross-sectional studies), reflective of the predetermined scope of the filter. The second author extracted the following unique broad concepts related to mental health conditions in the healthcare worker records: occupational stress, psychological resilience, compassion fatigue and burnout. No unique concepts were extracted for people of the community.

Once the broad concepts had been determined, specific search terminology was adopted from the headings and free-text terms present in the reviewed records, using a pragmatic approach to balance recall and specificity. The two authors of this paper each independently developed a search strategy using the extracted headings and free-text terms, combining the search terms with database- and platform-appropriate syntax and Boolean operators, and refining the search iteratively based on reviewing the records retrieved by particular term combinations. We opted to create the filter to search the titles and abstracts only because we found that many of the author-supplied keywords already appeared in the title and abstract. We wanted to increase the precision (reduce the amount of noise) and reduce the yield (the number of articles to screen) during the validation process.

### Validation Methods

The targets for the validation of a filter vary among researchers. We adopted a target recall of 90% as proposed by multiple studies (16, 18, 19). We assessed the filter's internal validity by calculating recall against the development sets for each population. We assessed the records for the missed references and included additional terms into the filter to improve recall, after which the calculation was redone. We then assessed the filter's external validity by calculating its recall against the validation set.

### Development of Precision Test Set

Finally, we assessed the precision of the filter by testing it in combination with the following topics: (i) children/adolescents and earthquakes; (ii) children/adolescents and Ebola outbreaks; (iii) healthcare workers and earthquakes; and (iv) healthcare workers and Ebola outbreaks. See Table 1 for the precision test search strings.

**Table 1:** Precision search test strings

Concept	Children and Adolescents	Healthcare Workers	Earthquakes	Ebola Virus Outbreaks
String	Child/or Adolescent/or (adolescen* or boy? or child or children or girl? or highschool* or high school* or juvenile? or kindergarten* or middle school* or preschool* or teen* or toddler? or tween* or youth).ti,ab.	exp Health Personnel/or exp Nurses/or exp Patient Care Team/or (allergist? or anatomist? or anesthetist? or anesthesiologist? or audiologist? or cardiologist? or clinician? or counsellor? or dermatologist? or dietitian? or doctor? or endocrinologist? or gastroenterologist? or geriatrician? or hospitalist? or nephrologist? or neurologist? or nurse? or nutritionist? or oncologist? or ophthalmologist? or pathologist? or pediatrician? or pharmacist? or physiatrist? or physician? or physiotherapist? or practitioner? or psychologist? or psychiatrist? or pulmonologist? or radiologist? or rheumatologist? or social worker? or surgeon? or urologist? or therapist?).ti,ab or ((allied-health or allied health or health or health-care or healthcare or hospital? or medical or nursing) adj2 (employee? or manager? or personnel or staff or team? or work*)).ti,ab.	Earthquakes/or earthquake?.ti,ab	Ebolavirus/or Hemorrhagic Fever, Ebola/or ebola*.ti,ab.

Key: ‘ti,ab’ refers to a search of the title and abstract fields. ‘/’ is indicative of a subject heading. ‘exp’ indicates explosion of the narrower terms beneath a heading. ‘*’ is used to substitute for zero or more characters. ‘?’ is used to substitute for zero or one character. ‘adjN’ is used for searching words that appear within N words of each other.

These strings are derived from one author's search practice and are not themselves validated; they were used simply to assess the precision of the proposed search filter in various contexts. Children and healthcare workers were selected because they represent subpopulations of common interest in discussion of the mental health impact of public health emergencies. Ebola and earthquakes were selected because they represent two different types of emergency (disease and natural disaster) and we wanted to avoid the problem of a filter too specific to the COVID-19 context that we noted in our search for existing filters. We also limited the results for all four scenarios to English-language articles published since 2019 as a convenience limit. All searches were conducted in Ovid MEDLINE.

## RESULTS

After developing the filter as described in the Methods, we then tested the two filters for acute mental health in people in the community and acute mental health for healthcare workers by assessing its recall against the development sets. The filter developed by the first author had a recall of 99% (102 out of 103 articles) against the development set for people in the community and 86% (137 out of 160 articles) against the development set for healthcare workers. The filter developed by the second author had a recall of 95% (98 out of 103 articles) against the development set for people in the community and 94% (151 out of 160 articles) against the development set for healthcare workers. After comparing the filters and assessing the records of the missed articles in each set, we determined that there were sufficient overlaps between the two search strategies to merit combining them into one filter.

Refer to Appendix A to review the initial search strategies developed by the two authors. The combined final filter is presented in Table 2. We then ran this filter against the development and validation sets for each population to assess internal and external validity. The results are summarized in Table 3.

**Table 2:** Combined final filter.

Line	String	Notes
1	Affective Symptoms/OR exp Behavioral Symptoms/OR exp Emotions/OR exp Mental Fatigue/OR Mental Health/OR Mental Health Services/OR exp Morale/OR exp Occupational Stress/OR Resilience, Psychological/OR exp Stress, Psychological/	General MeSH terms related to mental health symptoms
2	(exp Mental Disorders/OR exp Self-Injurious Behavior/) AND (Acute Disease/OR Cross-Sectional Studies/OR Crisis Intervention/OR Early Diagnosis/OR Incidence/OR Prevalence/)	Broader mental health MeSH combined with acute terminology; this was done to avoid retrieving material related to pre-existing mental health conditions.
3	((behavior* OR behaviour* OR emotion* OR mental OR psych*) adj3 (burnout OR burn out* OR distress* OR fatigue? OR impact? OR presentation? OR resilien* OR risk? OR symptom? OR wellbeing OR well-being OR wellness)).ti,ab.	General free-text terminology related to current behavioural/emotional distress/presentation
4	(((employment* OR job? OR job-related OR occupation* OR personal OR work OR workplace OR work-related) adj3 (distress* OR stress* OR resilience OR wellbeing OR well-being OR wellness)) OR compassion fatigue).ti,ab.	Terminology related to workplace or personal stress/resilience
5	((anxiet* OR anxious* OR burnout OR burn* out OR compassion fatigue OR demorali* OR depress* OR distress OR emotion* OR externalising OR externalizing OR fear OR grief OR grieving OR internalising OR internalizing OR mental* OR morale OR moral injur* OR overwhelm* OR panic OR peritrauma* OR peritrauma* OR phobi$2 OR psychiatr* OR psycho* OR posttrauma* OR post-trauma* OR post trauma* OR PTSD OR PTSS OR resilien* OR rumination OR somati* OR stress OR suicid* OR trauma* OR worry OR worrie$1 OR ((emotional* OR level? OR occupational OR perceive? OR perception* OR moral OR perception OR perceived OR psychological* OR work OR work-related) adj3 (distress OR stress*)) OR ((mental* OR emotional OR psychological*) adj3 (disorder* OR distress* OR disfunction* OR dysfunction* OR health OR well*)) OR ((emotional* OR health* OR job OR mental OR occupational OR professional OR psychological*) adj3 (resilien* OR wellness)) OR (emotion* adj2 (experienc* OR mindset OR negative*))) adj3 (acute OR behavio?r? OR burden? OR concomitant? OR concern? OR complain* OR condition? OR consequence? OR crossection* OR cross-section* OR current* OR detect* OR develop* OR difficulties OR during OR effect? OR elevated OR exacerbate? OR frequency OR heightened OR immediate* OR impact? OR incidence OR level? OR morbidity OR new-onset OR onset OR outcome? OR predict* OR present* OR prevalen* OR probable OR problem? OR rate? OR reaction? OR resilience OR responses OR score? OR screen* OR self-assess* OR self-report* OR sequela* OR short-term OR short term OR sign? OR states OR status OR subjective OR surveillance OR symptom* OR trend?)).ti,ab.	Broad free-text emotional and behavioral symptom terminology in combination with broad free-text acute situation terminology
6	1 or 2 or 3 or 4 or 5	Combination of all lines

Key: ‘ti,ab’ refers to a search of the title and abstract fields. ‘/’ is indicative of a subject heading. ‘exp’ indicates explosion of the narrower terms beneath a heading. ‘*’ is used to substitute for zero or more characters. ‘?’ is used to substitute for zero or one character. ‘$N’ is used to substitute for zero to N characters. ‘adjN’ is used for searching words that appear within N words of each other.

**Table 3:** Results of internal and external validity testing.

	People in the community	Healthcare workers
Development set	100% (103 of 103 retrieved)	95.6% (152 of 159 retrieved)
Validation set	100% (103 of 103 retrieved)	96.3% (154 of 160 retrieved

In each precision test scenario (summarized in Table 4), the precision was well above the 45% minimum proposed by Avau et al (17).

**Table 4:** Results of precision tests

Scenario	Result retrieved	Results relevant	Precision
Earthquakes and children/adolescents	292	274	94%
Ebola outbreaks and children/adolescents	33	26	81%
Earthquakes and healthcare workers	116	94	81%
Ebola outbreaks and healthcare workers	78	62	79%

## DISCUSSION

Developing searches related to mental health concerns outside of specific disorder is challenging because of the variety of terminology used to describe affective symptoms, which complicates comprehensive retrieval. This is the first validated filter to retrieve literature about acute mental health concerns in public health emergencies using MEDLINE (Ovid) to our knowledge. The filter was developed in accordance with the search filter development methodology using a gold standard suggested by Ayiku et al (14). It can be used and adapted to retrieve emerging scientific literature on acute mental health for any population group for any public health emergency.

The filter demonstrates very strong recall against our gold standard set and good precision in testing across topic areas and population groups, exceeding in all cases the 45% precision recommended by Avau et al (17) and the 90% target for recall proposed by multiple authors (16, 18, 19). While the internal and external validity of the filter for articles related to people in the community was 100% (see Table 3), both internal and external validity were slightly lower (95.6% and 96.3% respectively) for healthcare workers. We found that some studies in the sets for this population were broader in scope and used language less specific to mental health concerns in the abstract, such as referring to “challenges” encountered. However, all cases remained above the 90% recall standard proposed in the literature.

The combination of the filter with earthquakes and children/adolescents demonstrated the best performance at 94% precision. The other three combinations demonstrated slightly lower precision, each around 80%. There were two main causes of noise in all three scenarios: studies of disaster preparedness (eg. examining worries about a potential future event) and studies of long-term post-emergency impacts, which were outside the scope of acute mental health concerns. An additional cause of noise in the healthcare worker scenarios were studies about healthcare workers treating mental health concerns rather than experiencing mental health concerns themselves.

We recommend readers modify and adapt the terminologies used in this filter to their own particular contexts and informational needs during a specific public health emergency to increase precision. As proposed by Wilczyinski et. al. combining the contents with other search strategies (as we did for our testing) can also increase precision (31). For example, the string could be combined with specific populations, or with a string related to the public health emergency at issue. We anticipate that this filter would be appropriate for the rapid retrieval of evidence in an emergency situation, and that it can be adapted for broader knowledge synthesis efforts. In the former scenario we anticipate precision will be increased compared to our testing because literature on long-term impacts will not yet have emerged, and searchers could further increase precision by excluding disaster-preparedness literature if no secondary evidence is desired.

There were some limitations to our methodology. First, we based our search strategy on a definition of acute mental health based on two resources (DSM-5 and DynaMed), and that definition may not reflect the definition used by all professionals in the field of mental health. As such, the filter may unintentionally exclude studies relevant to alternative definitions. Second, we tested the precision of this search with only two contexts and two population groups. Other contexts and population groups may require additional terminologies that we may have unintendedly overlooked. Other disasters such as wildfires, hurricanes, biochemical attacks, political violence, wars, etc. and population groups such as seniors, vulnerable populations, etc. may experience different mental health symptoms that require different search terms. Additionally, searches involving healthcare workers as a population are challenging, not just in the case of this filter but more broadly, as it is difficult to isolate studies of their health concerns from studies of health concerns that they treat in other populations. Another limitation to this project is that the precision tests contained some convenience limitations that may have skewed the results. One final limitation to this project is that this filter is non-specific and could be too broad for researchers interested in specific symptoms such as traumatic stress, anxiety, depression, burnout, prolonged grief, etc. However, there may be instances where there might be a paucity of research in an emerging context and the title and abstract field might be too specific. In those cases, the users may consider applying the keywords in the filter to other indexes such as keyword (.kw) or keyword phase (.kf) to increase the filter's sensitivity.

This filter is designed to be used by clinicians, librarians, policymakers, or public health professionals to rapidly identify the best evidence on a key topic common to emergency situations. It is hoped that this initiative will encourage other professionals sourcing and developing search strategies in the scientific literature to develop more refined searches for various topics during public health emergencies.

## Data Availability

Data associated with this article are available on Open Science Framework at https://osf.io/nd28u/.
