# Supplementary material for: Acute mental health concerns in emergency settings: development and validation of an Ovid MEDLINE search filter
Source: J Med Libr Assoc. 2025 Aug 1;113(3):195–203. doi: 10.5195/jmla.2025.2081 (PMC12369960; doi:10.5195/jmla.2025.2081)
Supplement: Supplementary file 1 — Appendix A [file jmla-113-3-195-s01.docx]

**Appendix A: Precision Test Filters**

**Precision Test One: Children and Earthquakes**

| 1 | Affective Symptoms/ or exp Behavioral Symptoms/ or exp Emotions/ or exp Mental Fatigue/ or Mental Health/ or Mental Health Services/ or exp Morale/ or exp Occupational Stress/ or Resilience, Psychological/ or exp Stress, Psychological/ |
| --- | --- |
| 2 | (exp Mental Disorders/ or exp Self-Injurious Behavior/) and (Acute Disease/ or Cross-Sectional Studies/ or Crisis Intervention/ or Early Diagnosis/ or Incidence/ or Prevalence/) |
| 3 | ((behavior* or behaviour* or emotion* or mental or psych*) adj3 (burnout or burn out* or distress* or fatigue? or impact? or presentation? or resilien* or risk? or symptom? or wellbeing or well-being or wellness)).ti,ab. |
| 4 | (((employment* or job? or job-related or occupation* or personal or work or workplace or work-related) adj3 (distress* or stress* or resilience or wellbeing or well-being or wellness)) or burnout or compassion fatigue).ti,ab. |
| 5 | ((anxiet* or anxious* or burnout or burn* out or compassion fatigue or demorali* or depress* or distress or emotion* or externalising or externalizing or fear or grief or grieving or internalising or internalizing or mental* or morale or moral injur* or overwhelm* or panic or peritrauma* or peri-trauma* or phobi$2 or psychiatr* or psycho* or posttrauma* or post-trauma* or post trauma* or PTSD or PTSS or resilien* or rumination or somati* or stress or suicid* or trauma* or worry or worrie$1 or ((emotional* or level? or occupational or perceive? or perception* or moral or perception or perceived or psychological* or work or work-related) adj3 (distress or stress*)) or ((mental* or emotional or psychological*) adj3 (disorder* or distress* or disfunction* or dysfunction* or health or well*)) or ((emotional* or health* or job or mental or occupational or professional or psychological*) adj3 (resilien* or wellness)) or (emotion* adj2 (experienc* or mindset or negative*))) adj3 (acute or behavio?r? or burden? or concomitant? or concern? or complain* or condition? or consequence? or crossection* or cross-section* or current* or detect* or develop* or difficulties or during or effect? or elevated or exacerbate? or frequency or heightened or immediate* or impact? or incidence or level? or mobidity or new-onset or onset or outcome? or predict* or present* or prevalen* or probable or problem? or rate? or reaction? or resilience or responses or score? or screen* or self-assess* or self-report* or sequela* or short-term or short term or sign? or states or status or subjective or surveillance or symptom* or trend?)).ti,ab. |
| 6 | 1 or 2 or 3 or 4 or 5 |
| 7 | Earthquakes/ |
| 8 | earthquake?.ti,ab. |
| 9 | 7 or 8 |
| 10 | Child/ or Adolescent/ |
| 11 | (adolescen* or boy? or child or children or girl? or highschool* or high school* or juvenile? or kindergarten* or middle school* or preschool* or teen* or toddler? or tween* or youth).ti,ab. |
| 12 | 10 or 11 |
| 13 | 6 and 9 and 12 |
| 14 | limit 13 to (english language and yr="2019 -Current") |

**Precision Test Two: Children and Ebolavirus**

| 1 | Affective Symptoms/ or exp Behavioral Symptoms/ or exp Emotions/ or exp Mental Fatigue/ or Mental Health/ or Mental Health Services/ or exp Morale/ or exp Occupational Stress/ or Resilience, Psychological/ or exp Stress, Psychological/ |
| --- | --- |
| 2 | (exp Mental Disorders/ or exp Self-Injurious Behavior/) and (Acute Disease/ or Cross-Sectional Studies/ or Crisis Intervention/ or Early Diagnosis/ or Incidence/ or Prevalence/) |
| 3 | ((behavior* or behaviour* or emotion* or mental or psych*) adj3 (burnout or burn out* or distress* or fatigue? or impact? or presentation? or resilien* or risk? or symptom? or wellbeing or well-being or wellness)).ti,ab. |
| 4 | (((employment* or job? or job-related or occupation* or personal or work or workplace or work-related) adj3 (distress* or stress* or resilience or wellbeing or well-being or wellness)) or burnout or compassion fatigue).ti,ab. |
| 5 | ((anxiet* or anxious* or burnout or burn* out or compassion fatigue or demorali* or depress* or distress or emotion* or externalising or externalizing or fear or grief or grieving or internalising or internalizing or mental* or morale or moral injur* or overwhelm* or panic or peritrauma* or peri-trauma* or phobi$2 or psychiatr* or psycho* or posttrauma* or post-trauma* or post trauma* or PTSD or PTSS or resilien* or rumination or somati* or stress or suicid* or trauma* or worry or worrie$1 or ((emotional* or level? or occupational or perceive? or perception* or moral or perception or perceived or psychological* or work or work-related) adj3 (distress or stress*)) or ((mental* or emotional or psychological*) adj3 (disorder* or distress* or disfunction* or dysfunction* or health or well*)) or ((emotional* or health* or job or mental or occupational or professional or psychological*) adj3 (resilien* or wellness)) or (emotion* adj2 (experienc* or mindset or negative*))) adj3 (acute or behavio?r? or burden? or concomitant? or concern? or complain* or condition? or consequence? or crossection* or cross-section* or current* or detect* or develop* or difficulties or during or effect? or elevated or exacerbate? or frequency or heightened or immediate* or impact? or incidence or level? or mobidity or new-onset or onset or outcome? or predict* or present* or prevalen* or probable or problem? or rate? or reaction? or resilience or responses or score? or screen* or self-assess* or self-report* or sequela* or short-term or short term or sign? or states or status or subjective or surveillance or symptom* or trend?)).ti,ab. |
| 6 | 1 or 2 or 3 or 4 or 5 |
| 7 | Ebolavirus/ or Hemorrhagic Fever, Ebola/ |
| 8 | ebola*.ti,ab. |
| 9 | 7 or 8 |
| 10 | Child/ or Adolescent/ |
| 11 | (adolescen* or boy? or child or children or girl? or highschool* or high school* or juvenile? or kindergarten* or middle school* or preschool* or teen* or toddler? or tween* or youth).ti,ab. |
| 12 | 10 or 11 |
| 13 | 6 and 9 and 12 |
| 14 | limit 13 to (english language and yr="2019 -Current") |

**Precision Test Three: Healthcare Professionals and Earthquakes**

| 1 | Affective Symptoms/ or exp Behavioral Symptoms/ or exp Emotions/ or exp Mental Fatigue/ or Mental Health/ or Mental Health Services/ or exp Morale/ or exp Occupational Stress/ or Resilience, Psychological/ or exp Stress, Psychological/ |
| --- | --- |
| 2 | (exp Mental Disorders/ or exp Self-Injurious Behavior/) and (Acute Disease/ or Cross-Sectional Studies/ or Crisis Intervention/ or Early Diagnosis/ or Incidence/ or Prevalence/) |
| 3 | ((behavior* or behaviour* or emotion* or mental or psych*) adj3 (burnout or burn out* or distress* or fatigue? or impact? or presentation? or resilien* or risk? or symptom? or wellbeing or well-being or wellness)).ti,ab. |
| 4 | (((employment* or job? or job-related or occupation* or personal or work or workplace or work-related) adj3 (distress* or stress* or resilience or wellbeing or well-being or wellness)) or burnout or compassion fatigue).ti,ab. |
| 5 | ((anxiet* or anxious* or burnout or burn* out or compassion fatigue or demorali* or depress* or distress or emotion* or externalising or externalizing or fear or grief or grieving or internalising or internalizing or mental* or morale or moral injur* or overwhelm* or panic or peritrauma* or peri-trauma* or phobi$2 or psychiatr* or psycho* or posttrauma* or post-trauma* or post trauma* or PTSD or PTSS or resilien* or rumination or somati* or stress or suicid* or trauma* or worry or worrie$1 or ((emotional* or level? or occupational or perceive? or perception* or moral or perception or perceived or psychological* or work or work-related) adj3 (distress or stress*)) or ((mental* or emotional or psychological*) adj3 (disorder* or distress* or disfunction* or dysfunction* or health or well*)) or ((emotional* or health* or job or mental or occupational or professional or psychological*) adj3 (resilien* or wellness)) or (emotion* adj2 (experienc* or mindset or negative*))) adj3 (acute or behavio?r? or burden? or concomitant? or concern? or complain* or condition? or consequence? or crossection* or cross-section* or current* or detect* or develop* or difficulties or during or effect? or elevated or exacerbate? or frequency or heightened or immediate* or impact? or incidence or level? or mobidity or new-onset or onset or outcome? or predict* or present* or prevalen* or probable or problem? or rate? or reaction? or resilience or responses or score? or screen* or self-assess* or self-report* or sequela* or short-term or short term or sign? or states or status or subjective or surveillance or symptom* or trend?)).ti,ab. |
| 6 | 1 or 2 or 3 or 4 or 5 |
| 7 | Earthquakes/ |
| 8 | earthquake?.ti,ab. |
| 9 | 7 or 8 |
| 10 | exp Health Personnel/ or exp Nurses/ or exp Patient Care Team/ |
| 11 | (allergist? or anatomist? or anesthetist? or anesthesiologist? or audiologist? or cardiologist? or clinician? or counsellor? or dermatologist? or dietitian? or doctor? or endocrinologist? or gastroenterologist? or geriatrician? or hospitalist? or nephrologist? or neurologist? or nurse? or nutritionist? or oncologist? or ophthalmologist? or pathologist? or pediatrician? or pharmacist? or physiatrist? or physician? or physiotherapist? or practitioner? or psychologist? or psychiatrist? or pulmonologist? or radiologist? or rheumatologist? or social worker? or surgeon? or urologist? or therapist?).ti,ab. |
| 12 | ((allied-health or allied health or health or health-care or healthcare or hospital? or medical or nursing) adj2 (employee? or manager? or personnel or staff or team? or work*)).ti,ab. |
| 13 | 10 or 11 or 12 |
| 14 | 6 and 9 and 13 |
| 15 | limit 14 to (english language and yr="2019 -Current") |

**Precision Test Four: Healthcare Professionals and Ebolavirus**

| 1 | Affective Symptoms/ or exp Behavioral Symptoms/ or exp Emotions/ or exp Mental Fatigue/ or Mental Health/ or Mental Health Services/ or exp Morale/ or exp Occupational Stress/ or Resilience, Psychological/ or exp Stress, Psychological/ |
| --- | --- |
| 2 | (exp Mental Disorders/ or exp Self-Injurious Behavior/) and (Acute Disease/ or Cross-Sectional Studies/ or Crisis Intervention/ or Early Diagnosis/ or Incidence/ or Prevalence/) |
| 3 | ((behavior* or behaviour* or emotion* or mental or psych*) adj3 (burnout or burn out* or distress* or fatigue? or impact? or presentation? or resilien* or risk? or symptom? or wellbeing or well-being or wellness)).ti,ab. |
| 4 | (((employment* or job? or job-related or occupation* or personal or work or workplace or work-related) adj3 (distress* or stress* or resilience or wellbeing or well-being or wellness)) or burnout or compassion fatigue).ti,ab. |
| 5 | ((anxiet* or anxious* or burnout or burn* out or compassion fatigue or demorali* or depress* or distress or emotion* or externalising or externalizing or fear or grief or grieving or internalising or internalizing or mental* or morale or moral injur* or overwhelm* or panic or peritrauma* or peri-trauma* or phobi$2 or psychiatr* or psycho* or posttrauma* or post-trauma* or post trauma* or PTSD or PTSS or resilien* or rumination or somati* or stress or suicid* or trauma* or worry or worrie$1 or ((emotional* or level? or occupational or perceive? or perception* or moral or perception or perceived or psychological* or work or work-related) adj3 (distress or stress*)) or ((mental* or emotional or psychological*) adj3 (disorder* or distress* or disfunction* or dysfunction* or health or well*)) or ((emotional* or health* or job or mental or occupational or professional or psychological*) adj3 (resilien* or wellness)) or (emotion* adj2 (experienc* or mindset or negative*))) adj3 (acute or behavio?r? or burden? or concomitant? or concern? or complain* or condition? or consequence? or crossection* or cross-section* or current* or detect* or develop* or difficulties or during or effect? or elevated or exacerbate? or frequency or heightened or immediate* or impact? or incidence or level? or mobidity or new-onset or onset or outcome? or predict* or present* or prevalen* or probable or problem? or rate? or reaction? or resilience or responses or score? or screen* or self-assess* or self-report* or sequela* or short-term or short term or sign? or states or status or subjective or surveillance or symptom* or trend?)).ti,ab. |
| 6 | 1 or 2 or 3 or 4 or 5 |
| 7 | Ebolavirus/ or Hemorrhagic Fever, Ebola/ |
| 8 | ebola*.ti,ab. |
| 9 | 7 or 8 |
| 10 | exp Health Personnel/ or exp Nurses/ or exp Patient Care Team/ |
| 11 | (allergist? or anatomist? or anesthetist? or anesthesiologist? or audiologist? or cardiologist? or clinician? or counsellor? or dermatologist? or dietitian? or doctor? or endocrinologist? or gastroenterologist? or geriatrician? or hospitalist? or nephrologist? or neurologist? or nurse? or nutritionist? or oncologist? or ophthalmologist? or pathologist? or pediatrician? or pharmacist? or physiatrist? or physician? or physiotherapist? or practitioner? or psychologist? or psychiatrist? or pulmonologist? or radiologist? or rheumatologist? or social worker? or surgeon? or urologist? or therapist?).ti,ab. |
| 12 | ((allied-health or allied health or health or health-care or healthcare or hospital? or medical or nursing) adj2 (employee? or manager? or personnel or staff or team? or work*)).ti,ab. |
| 13 | 10 or 11 or 12 |
| 14 | 6 and 9 and 13 |
| 15 | limit 14 to (english language and yr="2019 -Current") |
