# Supplementary material for: Acute mental health concerns in emergency settings: development and validation of an Ovid MEDLINE search filter
Source: J Med Libr Assoc. 2025 Aug 1;113(3):195–203. doi: 10.5195/jmla.2025.2081 (PMC12369960; doi:10.5195/jmla.2025.2081)
Supplement: Supplementary file 2 — Appendix B [file jmla-113-3-195-s02.docx]

**Appendix B: Review Articles for People in the Community**

1. Yunitri N, Chu H, Kang XL, et al. Global prevalence and associated risk factors of posttraumatic stress disorder during COVID-19 pandemic: A meta-analysis. *Int J Nurs Stud*. 2022;126:104136. doi:[10.1016/j.ijnurstu.2021.104136](https://doi.org/10.1016/j.ijnurstu.2021.104136)

2. Mental Health, Brain Health and Substance Use (MSD), WHO Headquarters (HQ). Mental Health and COVID-19: Early evidence of the pandemic’s impact: Scientific brief, 2 March 2022. Published online March 2, 2022. <https://www.who.int/publications/i/item/WHO-2019-nCoV-Sci_Brief-Mental_health-2022.1>

3. Golitaleb M, Mazaheri E, Bonyadi M, Sahebi A. Prevalence of Post-traumatic Stress Disorder After Flood: A Systematic Review and Meta-Analysis. *Front Psychiatry*. 2022;13:890671. doi:[10.3389/fpsyt.2022.890671](https://doi.org/10.3389/fpsyt.2022.890671)

4. Brooks SK, Patel SS. Psychological Consequences of the Flint Water Crisis: A Scoping Review - Erratum. *Disaster Med Public Health Prep*. 2022;16(3):1282. doi:[10.1017/dmp.2021.234](https://doi.org/10.1017/dmp.2021.234)

5. Zhou Y, Sun Z, Wang Y, et al. The prevalence of PTSS under the influence of public health emergencies in last two decades: A systematic review and meta-analysis. *Clin Psychol Rev*. 2021;83:101938. doi:[10.1016/j.cpr.2020.101938](https://doi.org/10.1016/j.cpr.2020.101938)

6. Stein J, Niemeyer H, Meyer C, et al. Posttraumatic stress in adult civilians exposed to violent conflict, war and associated human rights abuses in the Eastern Mediterranean Region: A systematic review and meta-analysis. *J Affect Disord*. 2021;294:605-627. doi:[10.1016/j.jad.2021.06.042](https://doi.org/10.1016/j.jad.2021.06.042)

7. Sharpe I, Davison CM. Climate change, climate-related disasters and mental disorder in low- and middle-income countries: a scoping review. *BMJ Open*. 2021;11(10):e051908. doi:[10.1136/bmjopen-2021-051908](https://doi.org/10.1136/bmjopen-2021-051908)

8. Santabárbara J, Lasheras I, Lipnicki DM, et al. Prevalence of anxiety in the COVID-19 pandemic: An updated meta-analysis of community-based studies. *Prog Neuropsychopharmacol Biol Psychiatry*. 2021;109:110207. doi:[10.1016/j.pnpbp.2020.110207](https://doi.org/10.1016/j.pnpbp.2020.110207)

9. McKinzie AE, Clay-Warner J. The Gendered Effect of Disasters on Mental Health: A Systematic Review. *International Journal of Mass Emergencies & Disasters*. 2021;39(2):227-262. doi:[10.1177/028072702103900202](https://doi.org/10.1177/028072702103900202)

10. Cénat JM, Blais-Rochette C, Kokou-Kpolou CK, et al. Prevalence of symptoms of depression, anxiety, insomnia, posttraumatic stress disorder, and psychological distress among populations affected by the COVID-19 pandemic: A systematic review and meta-analysis. *Psychiatry Res*. 2021;295:113599. doi:[10.1016/j.psychres.2020.113599](https://doi.org/10.1016/j.psychres.2020.113599)

11. Ni MY, Kim Y, McDowell I, et al. Mental health during and after protests, riots and revolutions: A systematic review. *Aust N Z J Psychiatry*. 2020;54(3):232-243. doi:[10.1177/0004867419899165](https://doi.org/10.1177/0004867419899165)

12. Luo Y, Chua CR, Xiong Z, Ho RC, Ho CSH. A Systematic Review of the Impact of Viral Respiratory Epidemics on Mental Health: An Implication on the Coronavirus Disease 2019 Pandemic. *Front Psychiatry*. 2020;11:565098. doi:[10.3389/fpsyt.2020.565098](https://doi.org/10.3389/fpsyt.2020.565098)

13. Kerdemelidis M, Reid M, Planning & Funding, Canterbury District Health Board, Christchurch, New Zealand. Wellbeing recovery after mass shootings: information for the response to the Christchurch mosque attacks 2019: Rapid literature review. Published online 2019. <https://www.cdhb.health.nz/wp-content/uploads/5fe3e197-rapid-literature-review-cdhb-response-christchurch-mosque-attacks-2019.pdf>

14. El Baba R, Colucci E. Post-traumatic stress disorders, depression, and anxiety in unaccompanied refugee minors exposed to war-related trauma: a systematic review. *International Journal of Culture and Mental Health*. 2018;11(2):194-207. doi:[10.1080/17542863.2017.1355929](https://doi.org/10.1080/17542863.2017.1355929)

15. Lowe SR, Galea S. The Mental Health Consequences of Mass Shootings. *Trauma Violence Abuse*. 2017;18(1):62-82. doi:[10.1177/1524838015591572](https://doi.org/10.1177/1524838015591572)

16. Paz García-Vera M, Sanz J, Gutiérrez S. A Systematic Review of the Literature on Posttraumatic Stress Disorder in Victims of Terrorist Attacks. *Psychol Rep*. 2016;119(1):328-359. doi:[10.1177/0033294116658243](https://doi.org/10.1177/0033294116658243)

17. Dai W, Chen L, Lai Z, Li Y, Wang J, Liu A. The incidence of post-traumatic stress disorder among survivors after earthquakes:a systematic review and meta-analysis. *BMC Psychiatry*. 2016;16:188. doi:[10.1186/s12888-016-0891-9](https://doi.org/10.1186/s12888-016-0891-9)

18. Fernandez A, Black J, Jones M, et al. Flooding and mental health: a systematic mapping review. *PLoS One*. 2015;10(4):e0119929. doi:[10.1371/journal.pone.0119929](https://doi.org/10.1371/journal.pone.0119929)

19. Shultz JM, Thoresen S, Flynn BW, et al. Multiple vantage points on the mental health effects of mass shootings. *Curr Psychiatry Rep*. 2014;16(9):469. doi:[10.1007/s11920-014-0469-5](https://doi.org/10.1007/s11920-014-0469-5)

20. North CS, Pfefferbaum B. Mental health response to community disasters: a systematic review. *JAMA*. 2013;310(5):507-518. doi:[10.1001/jama.2013.107799](https://doi.org/10.1001/jama.2013.107799)

21. Peltonen K, Punamäki RL. Preventive interventions among children exposed to trauma of armed conflict: a literature review. *Aggress Behav*. 2010;36(2):95-116. doi:[10.1002/ab.20334](https://doi.org/10.1002/ab.20334)
