# Supplementary material for: Acute mental health concerns in emergency settings: development and validation of an Ovid MEDLINE search filter
Source: J Med Libr Assoc. 2025 Aug 1;113(3):195–203. doi: 10.5195/jmla.2025.2081 (PMC12369960; doi:10.5195/jmla.2025.2081)
Supplement: Supplementary file 3 — Appendix C [file jmla-113-3-195-s03.docx]

**Appendix C: Review Articles for Healthcare Workers**

1. Tahernejad S, Ghaffari S, Ariza-Montes A, Wesemann U, Farahmandnia H, Sahebi A. Post-traumatic stress disorder in medical workers involved in earthquake response: A systematic review and meta-analysis. *Heliyon*. 2023;9(1):e12794. doi:[10.1016/j.heliyon.2023.e12794](https://doi.org/10.1016/j.heliyon.2023.e12794)

2. Palmer J, Ku M, Wang H, et al. Public health emergency and psychological distress among healthcare workers: a scoping review. *BMC Public Health*. 2022;22(1):1396. doi:[10.1186/s12889-022-13761-1](https://doi.org/10.1186/s12889-022-13761-1)

3. Mental Health, Brain Health and Substance Use (MSD), WHO Headquarters (HQ). Mental Health and COVID-19: Early evidence of the pandemic’s impact: Scientific brief, 2 March 2022. Published online March 2, 2022. <https://www.who.int/publications/i/item/WHO-2019-nCoV-Sci_Brief-Mental_health-2022.1>

4. Hill JE, Harris C, Danielle L C, et al. The prevalence of mental health conditions in healthcare workers during and after a pandemic: Systematic review and meta-analysis. *J Adv Nurs*. 2022;78(6):1551-1573. doi:[10.1111/jan.15175](https://doi.org/10.1111/jan.15175)

5. Zhou Y, Sun Z, Wang Y, et al. The prevalence of PTSS under the influence of public health emergencies in last two decades: A systematic review and meta-analysis. *Clin Psychol Rev*. 2021;83:101938. doi:[10.1016/j.cpr.2020.101938](https://doi.org/10.1016/j.cpr.2020.101938)

6. Sun P, Wang M, Song T, et al. The Psychological Impact of COVID-19 Pandemic on Health Care Workers: A Systematic Review and Meta-Analysis. *Front Psychol*. 2021;12:626547. doi:[10.3389/fpsyg.2021.626547](https://doi.org/10.3389/fpsyg.2021.626547)

7. Salehi M, Amanat M, Mohammadi M, et al. The prevalence of post-traumatic stress disorder related symptoms in Coronavirus outbreaks: A systematic-review and meta-analysis. *J Affect Disord*. 2021;282:527-538. doi:[10.1016/j.jad.2020.12.188](https://doi.org/10.1016/j.jad.2020.12.188)

8. Sahebi A, Yousefi A, Abdi K, et al. The Prevalence of Post-traumatic Stress Disorder Among Health Care Workers During the COVID-19 Pandemic: An Umbrella Review and Meta-Analysis. *Front Psychiatry*. 2021;12:764738. doi:[10.3389/fpsyt.2021.764738](https://doi.org/10.3389/fpsyt.2021.764738)

9. Li Y, Scherer N, Felix L, Kuper H. Prevalence of depression, anxiety and post-traumatic stress disorder in health care workers during the COVID-19 pandemic: A systematic review and meta-analysis. *PLoS One*. 2021;16(3):e0246454. doi:[10.1371/journal.pone.0246454](https://doi.org/10.1371/journal.pone.0246454)

10. Hao Q, Wang D, Xie M, et al. Prevalence and Risk Factors of Mental Health Problems Among Healthcare Workers During the COVID-19 Pandemic: A Systematic Review and Meta-Analysis. *Front Psychiatry*. 2021;12:567381. doi:[10.3389/fpsyt.2021.567381](https://doi.org/10.3389/fpsyt.2021.567381)

11. Godfrey CM, Rodgers J, Pare GC, Alsius A, Ross-White A, Belbin S, Sears K. Healthcare Provider Burnout: A Rapid Scoping Review. Published online 2021. <https://www.mcmasterforum.org/docs/default-source/product-documents/rapid-responses/healthcare-provider-burnout.pdf?sfvrsn=70b2c7bb_5>

12. Chigwedere OC, Sadath A, Kabir Z, Arensman E. The Impact of Epidemics and Pandemics on the Mental Health of Healthcare Workers: A Systematic Review. *Int J Environ Res Public Health*. 2021;18(13):6695. doi:[10.3390/ijerph18136695](https://doi.org/10.3390/ijerph18136695)

13. Cénat JM, Blais-Rochette C, Kokou-Kpolou CK, et al. Prevalence of symptoms of depression, anxiety, insomnia, posttraumatic stress disorder, and psychological distress among populations affected by the COVID-19 pandemic: A systematic review and meta-analysis. *Psychiatry Res*. 2021;295:113599. doi:[10.1016/j.psychres.2020.113599](https://doi.org/10.1016/j.psychres.2020.113599)

14. Busch IM, Moretti F, Mazzi M, Wu AW, Rimondini M. What We Have Learned from Two Decades of Epidemics and Pandemics: A Systematic Review and Meta-Analysis of the Psychological Burden of Frontline Healthcare Workers. *Psychother Psychosom*. 2021;90(3):178-190. doi:[10.1159/000513733](https://doi.org/10.1159/000513733)

15. Al Falasi B, Al Mazrouei M, Al Ali M, et al. Prevalence and Determinants of Immediate and Long-Term PTSD Consequences of Coronavirus-Related (CoV-1 and CoV-2) Pandemics among Healthcare Professionals: A Systematic Review and Meta-Analysis. *Int J Environ Res Public Health*. 2021;18(4):2182. doi:[10.3390/ijerph18042182](https://doi.org/10.3390/ijerph18042182)

16. Stuijfzand S, Deforges C, Sandoz V, et al. Psychological impact of an epidemic/pandemic on the mental health of healthcare professionals: a rapid review. *BMC Public Health*. 2020;20(1):1230. doi:[10.1186/s12889-020-09322-z](https://doi.org/10.1186/s12889-020-09322-z)

17. Sriharan A, Ratnapalan S, Tricco AC, et al. Occupational Stress, Burnout, and Depression in Women in Healthcare During COVID-19 Pandemic: Rapid Scoping Review. *Front Glob Womens Health*. 2020;1:596690. doi:[10.3389/fgwh.2020.596690](https://doi.org/10.3389/fgwh.2020.596690)

18. Pollock A, Campbell P, Cheyne J, et al. Interventions to support the resilience and mental health of frontline health and social care professionals during and after a disease outbreak, epidemic or pandemic: a mixed methods systematic review. *Cochrane Database Syst Rev*. 2020;11(11):CD013779. doi:[10.1002/14651858.CD013779](https://doi.org/10.1002/14651858.CD013779)

19. Batra K, Singh TP, Sharma M, Batra R, Schvaneveldt N. Investigating the Psychological Impact of COVID-19 among Healthcare Workers: A Meta-Analysis. *Int J Environ Res Public Health*. 2020;17(23):9096. doi:[10.3390/ijerph17239096](https://doi.org/10.3390/ijerph17239096)

20. Lowell A, Suarez-Jimenez B, Helpman L, et al. 9/11-related PTSD among highly exposed populations: a systematic review 15 years after the attack. *Psychol Med*. 2018;48(4):537-553. doi:[10.1017/S0033291717002033](https://doi.org/10.1017/S0033291717002033)

21. Guilaran J, De Terte I, Kaniasty K, Stephens C. Psychological Outcomes in Disaster Responders: A Systematic Review and Meta-Analysis on the Effect of Social Support. *Int J Disaster Risk Sci*. 2018;9(3):344-358. doi:[10.1007/s13753-018-0184-7](https://doi.org/10.1007/s13753-018-0184-7)

22. Brooks SK, Dunn R, Amlôt R, Rubin GJ, Greenberg N. Social and occupational factors associated with psychological wellbeing among occupational groups affected by disaster: a systematic review. *Journal of Mental Health*. 2017;26(4):373-384. doi:[10.1080/09638237.2017.1294732](https://doi.org/10.1080/09638237.2017.1294732)
